# Investigating the usefulness of a metacognitive training group programme for schizophrenia

**DOI:** 10.1192/pb.bp.113.046037

**Published:** 2015-06

**Authors:** Lorna Jane Howe, Ian D. Brown

**Affiliations:** 1 Cambian Healthcare Ltd, Mansfield; 2 University of Sheffield, Sheffield

## Abstract

**Aims and method** To examine the usefulness of a cognitive-behavioural therapy-based group intervention, metacognitive training for schizophrenia (MCT), in a ‘real-world’ clinical setting. In total, 164 participants completed 327 questionnaires at the end of MCT group sessions; rating the perceived usefulness, helpfulness to recovery, change in knowledge and anxiety. Non-parametric statistical tests were used to analyse the data.

**Results** Participants indicated positive responses in terms of perceived usefulness, helpfulness to recovery and increased knowledge following group attendance. Significant positive correlations were found between: (a) usefulness and helpfulness to recovery, and (b) helpfulness to recovery and change in knowledge. There were significant negative correlations between: (a) usefulness and anxiety, and (b) helpfulness to recovery and anxiety.

**Clinical implications** The results suggest that MCT is a useful and effective evidence-based psychological intervention. It supports the use of cognitive-behavioural interventions in the treatment of individuals experiencing psychosis, although further evaluation is needed.

The need for psychological interventions for psychosis is increasingly recognised^1^ and cognitive–behavioural interventions have emerged as potentially effective approaches that should be considered in the treatment of schizophrenia.^2^ The rationale for such approaches stems from mounting evidence that cognitive biases may trigger or maintain symptoms in schizophrenia, especially rigidly held unusual beliefs, frequently described as delusions within the literature.^3^ Individuals with the diagnosis may show evidence of attributional biases, jumping to conclusions, bias against disconfirmatory evidence, overconfidence in errors and problems with theory of mind.^4^ In light of these findings, an evidence-based group cognitive training programme has been developed called metacognitive training for schizophrenia (MCT)^5^ (metacognitive can be defined as thinking about one’s own thinking). The programme aims to provide psychoeducation on cognitive biases to help alter individual’s current problem-solving repertoire and prevent relapse.^5,6^ Research has confirmed the feasibility of MCT and provided support for its efficacy in reducing positive symptoms and cognitive biases related to schizophrenia in 29 countries including Germany^7,8^ and Switzerland.^9,10^

However, although there is already support for the efficacy of MCT^7,8^ exploring its effectiveness within the local service was deemed important and, if necessary, adapting the service on the basis of feedback. This was reinforced by a health trust-wide initiative to promote clinical effectiveness through service-user feedback. It was considered important to examine individual sessions/modules as well as the broader programme experience (collapsing across attended modules). The open nature of the programme was such that participants could enter the programme at different points in the cycle: comparison of individual modules would show whether different entry-points to the programme were seen to be similarly useful/effective and identify particular foci for improvement. Three specific aims emerged from local service needs: to (a) explore the perceived utility and effectiveness of each MCT module, (b) explore the overall perceived utility and effectiveness of MCT group attendance (collapsing across sessions attended), and (c) examine the interrelationships between post-session ratings of perceived group utility, effectiveness and self-reported anxiety.

## Method

### Participants

Flyers inviting individuals to attend MCT sessions were disseminated to potentially suitable participants through professionals within the local community mental health team and acute in-patient ward. There were 164 participants; 105 attended only one session and 59 attended multiple sessions (mean 3.8 sessions, range 2–17). The mean age of participants was 42.7 (range 16–85); excluding 18 participants who did not give their age. Seventy respondents indicated they were male (43%) and 82 female (50%). Participants were not asked for details of their diagnosis in order to ensure the sessions were as non-invasive as possible.

### Procedure

At the end of each session, participants were given a questionnaire to rate the utility and effectiveness of the programme. Throughout eight cycles (64 sessions) of the group, 327 questionnaires were completed.

### Measures

The questionnaire was developed by the group facilitator (a clinical psychologist) based on the questionnaire used by the authors of MCT.^5^ It took approximately 5 min to complete and covered demographic information, questions that created a unique anonymised code to track participants through the programme and questions providing quantitative data on either a five-point or ten-point Likert scale (Appendix).

### Description of the programme

The MCT programme consisted of eight sessions on a weekly basis lasting approximately 45 min, facilitated by a clinical psychologist. There were different modules each week, which consisted of familiarising participants with the

**Table 1:** Programme content^11^

Module title	Target domain	Overview of exercises
Module 1a & 1b: Attribution – blaming and taking credit	Self-serving bias *v.* depressive attributional bias	Different reasons for positive and negative events are contemplated. Explanations that account for various causes are preferred to single explanations

Module 2a & 2b: Jumping to conclusions I	Jumping to conclusions/ liberal acceptance/bias against disconfirmatory evidence	Situations leading to rash decisions are discussed and disadvantages are emphasised. Disjointed pictures are shown that ultimately display objects

Module 3a & 3b: Changing beliefs	Bias against disconfirmatory evidence	Cartoon sequences are shown in backward order. Individuals learn to withhold strong judgements until adequate evidence has been collected

Module 4a & 4b: To empathise I	Theory of mind	Incomplete cartoon strips and pictures of human faces are presented, and participants are asked how the people depicted might feel. The first intuition is often wrong, showing that relying solely on facial expression can be misleading and multiple cues should be used

Module 5a & 5b: Memory	Overconfidence in errors	Factors that may promote or impair memory acquisition are discussed. Complex scenes are displayed with two typical elements each removed. Participants learn to differentiate between false and correct memories by their vividness

Module 6a & 6b: To empathise II	Theory of mind/need for closure	Different features guiding theory of mind are discussed. Cartoon sequences are presented, and the perspective of one of the characters is considered

Module 7a & 7b: Jumping to conclusions II	Jumping to conclusions/ liberal acceptance	Similar to module 2, the disadvantages of hasty decisions are outlined. Paintings are also displayed, and the correct title must be inferred from four options

Module 8a & 8b: Self-esteem and mood	Mood and self-esteem	Causes, symptoms, and treatment of depression are discussed. Typical depressive cognitive patterns are presented. Strategies to help enhance self-esteem and improve mood are also discussed

target domain (for example jumping to conclusions, attributional style), using everyday examples and illustrations projected from a computer screen. Two cycles of the programme were available; each cycle involved the same targets but with different group exercises. It was an open group that individuals could join at any time. The manual, modules and other resources were downloaded cost-free from www.uke.de/mct. Table 1 outlines the content of the group programme.^11^

## Results

Data was not normally distributed, therefore, non-parametric tests were used and the median and interquartile ranges (IQR) are reported throughout.

### Utility and effectiveness of each module

Table 2 shows the descriptive statistics for each module of the group programme; 266 out of 327 participants’ completed questionnaires were included; 61 were excluded because of missing data. Mann–Whitney *U*-tests were used to compare differences between ratings given for parallel sessions within each module. No significant differences were found; therefore, the two cycles of the programme were considered equivalent and the data was pooled.

As Table 2 shows, ratings for each module seemed fairly positive in terms of usefulness, change in knowledge and helpfulness to recovery. A Kruskal–Wallis test was conducted to investigate whether there were any significant differences in perceived utility and effectiveness between

**Table 2:** Median (interquartile range) ratings by module^a^

		Median (IQR)					
Module	*n*	Usefulness	Knowledge – prior	Knowledge – post	Change in knowledge	Helpfulness to recovery	Anxiety (session-end)
1	31	4.0 (3.0–5.0)	4.0 (1.0–6.0)	7.0 (4.0–9.0)	2.0 (0.0–4.0)	5.0 (3.0–10.0)	4.0 (1.0–6.0)

2	35	4.0 (3.0–5.0)	5.0 (3.0–7.0)	6.0 (5.0–8.0)	1.0 (0.0–3.0)	5.0 (3.0–9.0)	5.0 (1.0–7.0)

3	33	4.0 (3.5–4.5)	4.0 (1.0–6.0)	6.0 (5.0–8.0)	2.0 (0.5–4.0)	6.0 (4.5–9.5)	4.0 (1.0–6.0)

4	35	4.0 (3.0–4.0)	4.0 (1.0–5.0)	6.0 (5.0–8.0)	3.0 (1.0–4.0)	6.0 (4.0–7.0)	4.0 (1.0–8.0)

5	34	4.0 (3.0–4.25)	3.0 (1.0–6.25)	7.0 (4.0–7.0)	2.0 (1.0–3.88)	5.0 (2.0–7.0)	4.5 (1.0–6.25)

6	36	4.0 (3.25–5.0)	4.0 (1.25–7.0)	7.0 (5.0–9.0)	2.0 (1.0–4.0)	6.0 (3.0–7.0)	4.0 (1.0–6.0)

7	28	4.0 (3.0–4.0)	4.0 (1.0–7.75)	7.0 (4.25–8.75)	2.0 (1.0–3.75)	5.0 (4.0–7.0)	3.5 (1.0–5.0)

8	34	4.0 (3.0–5.0)	4.5 (2.0–6.25)	7.0 (3.75–9.25)	1.0 (0.0–3.0)	4.5 (2.0–8.0)	5.0 (1.75–8.0)

a. *n* represents the number of participants providing ratings for each module. Usefulness was rated on a five-point scale anchored at: 1, ‘unhelpful’ and 5, ‘very helpful’; knowledge, helpfulness to recovery and anxiety were all rated on a ten-point scale anchored at 1, ‘none at all’ and 10, ‘a great deal’.

modules. No significant differences were found on usefulness (*H* (*n* = 154) = 2.738, *P* = 0.908), change in knowledge (*H* (*n* = 157) = 11.558, *P* = 0.116), and helpfulness to recovery (*H* (*n* = 154) = 5.057 *P* = 0.653). To ensure that each rating was independent, only the first observation from each of the 59 individuals who attended more than one session was taken, and some individuals were excluded because of missing data. This suggested that ratings for each module were comparable and it was concluded that evaluating the group as a whole to evaluate the remaining aims of this paper was justifiable.

### Overall utility and effectiveness of group attendance

Subsequent analyses collapsed data across sessions to produce individual-level summaries. Thus, each unit of observation represents a separate individual, and each data-value represents the average score for that individual, across the sessions that they attended. In this way, all observations are independent and comparable, facilitating descriptive and inferential analyses of aggregated individual-level data that reflect the overall impact of group attendance.

On average, participants reported that sessions were fairly useful (median 4) and helped towards their recovery somewhat (median 4.5). Wilcoxon Signed Ranks tests were conducted for individual-average ratings of change in knowledge. Participants reported a highly significant increase in knowledge from pre-group (median 4.5) to post-group (median 5.5), *z* = –5.79, *P*<0.001.

Although there were overall (sample-level) changes in knowledge, inspection of individual change-scores seemed

**Table 3:** Spearman’s rho correlations for overall group ratings (*n* = 150)

	Helpfulness to recovery		Change in knowledge		Anxiety	
	*r*	*P*	*r*	*P*	*r*	*P*
Usefulness	0.288	<0.001	–0.053	0.514	–0.301	<0.001

Helpfulness to recovery			0.206	0.010	–0.194	0.018

Change in knowledge					–0.040	0.625

to show that some individuals reported no change or negative change between pre- and post-group. Specifically, 20% (32/159) reported zero or negative changes in knowledge. It was not possible to compute accurate reliable change estimates^12^ for knowledge items, as available estimates of test–retest reliability are conflated with intervention effects. Those who showed zero and negative changes would not be able to demonstrate reliable improvement in any analysis of reliable change.

### Interrelationships between group utility, effectiveness and self-reported anxiety

The Spearman’s rank order correlation coefficient (i.e. Spearman’s rho) was performed to explore the interrelationships between group ratings (Table 3). The Spearman’s rho revealed significant positive correlations between (a) usefulness and helpfulness to recovery and (b) helpfulness to recovery and change in knowledge. Furthermore, there were significant negative correlations between (a) usefulness and anxiety and (b) helpfulness to recovery and anxiety.

## Discussion

Participants indicated positive responses towards MCT in terms of perceived usefulness and helpfulness to recovery. Changes in outcome measures revealed an overall increase in knowledge following group attendance, although at an individual level some individuals did not report any increase in knowledge (this is discussed further in the Limitations section).

No particular sessions were perceived as more useful or effective than others. This supports the clinical application of all components of the programme and could be seen to support the open format of the group, since all entry-points are generally comparable in terms of utility.

It seems that the more useful participants found the group, the more they found it helpful towards their recovery – and vice versa. In keeping with the aims of the group, helpfulness to recovery was also positively correlated with change in knowledge. By increasing an individual’s awareness of cognitive biases and providing corrective experiences, it could be expected that an individual would report an increase in knowledge and related recovery (in terms of decreased symptoms).^5^ However, it is acknowledged that ‘recovery’ is a complex term and, although recovery from clinical symptoms can be seen as an outcome, individuals may continue to experience psychological distress while achieving ‘personal’ and ‘social’ recovery.^13,14^

Interestingly, self-reported anxiety was negatively correlated with perceived usefulness and helpfulness to recovery. This suggests that the more anxious participants were, the less useful and helpful towards their recovery the group was – and vice versa. This may have important clinical implications for future practice, which are discussed below.

### Limitations

Despite participants’ responses supporting the utility and effectiveness of MCT, which met various criteria for statistical significance, a number of limitations must be noted. The frequency of zero and negative individual-level change-scores suggests that some participants did not demonstrate knowledge improvements. From the available data it is unclear why this may be. It could be hypothesised that because the programme was an open group, establishing group cohesion was difficult. Therefore, although some individuals may have benefitted from the social processes of the group, some may have found the situation unhelpful and possibly anxiety-provoking. This may have had an impact on their ability to process and retain the information.

As participants were not specifically asked about their diagnosis, individuals who did not experience psychosis may have been included. As such, the programme content may not be suitable for those individuals and they may not have benefited from the group. Nevertheless, it is suggested that individuals with various mental health difficulties may benefit from MCT as the focus is on providing a neutral ‘common ground’ for discussing thinking styles, rather than individual symptoms.^11^ However, this remains to be investigated and was beyond the scope of this paper.

It is also noted that some participants only attended the group once or a few times. This may be a behavioural indication that the intervention was not working. On the other hand, it may indicate that individuals were in the process of recovery and felt they no longer needed MCT. There are also contextual issues to consider; for instance, those participants who were in-patients may have been discharged and reluctant to return to the group because they were feeling better or a desire to disassociate with the hospital environment.

The service-developed questionnaire also had a number of shortcomings that may have affected the results. Change-scores were based on retrospective measures that may have resulted in inaccurate estimates, or participants may have felt obliged to respond in accordance with perceived demand characteristics. Furthermore, the items do not map onto the specific targets of MCT, including the expected reduction of positive symptoms and cognitive biases. In addition, it was difficult to establish what ‘recovery’ meant to respondents and how they evaluated this. As mentioned above, recovery is a very complex and individual experience and the quantitative data did not capture this.

### Clinical implications

Despite the limitations, the results address the aims of the paper and suggest that MCT can provide a useful and effective evidence-based psychological intervention to participants within a local service. In addition to meeting local service needs, this paper contributes to the broader evidence base for MCT and supports the use of cognitive–behavioural interventions in the treatment of individuals experiencing psychosis.^2,7,8^

The results have provided some important insights that may help to inform future clinical practice. Correlations suggested that individuals may need support to manage their anxiety in order to facilitate the processing of programme content (for example using relaxation and ‘ice-breaker’ exercises at the beginning of sessions). It may also be useful for the group facilitator to have an open dialogue with participants about the effects of the group and recognise that not everyone may benefit from MCT. Furthermore, as some individuals did not appear to benefit from the group, more selective inclusion criteria may be needed (for example ensuring only individuals with a diagnosis of schizophrenia/psychosis are included). It may also be useful to implement the recently developed individualised MCT programme on a one-to-one basis with some clients who may not benefit from a group format.^15^

The MCT programme showed promising results in promoting knowledge and was helpful for recovery and therefore further evaluation of the MCT group programme is needed in the future. There were various design limitations of the questionnaire that would need to be addressed in order to improve future evaluation: (a) some participant demographics should be collected, including diagnosis, (b) measures of change should be taken before and after sessions in order to overcome problems with retrospective accounts, (c) items should map more tightly to the theoretical targets of MCT and could include objective tests (for example multiple-choice questions) *v.* subjective items that are more open to bias, (c) space for qualitative data should be provided under each question – particularly in relation to ‘recovery’ and what participants found helpful/unhelpful, and (d) questions about the impact of the facilitator’s style of delivery. This would allow exploration of what influences on outcomes relate to programme content or facilitator’s presentation skills.

## Appendix

### Questions on the evaulation form

**Table:**

How much knowledge did you have on the topic being covered prior to this session?									
1	2	3	4	5	6	7	8	9	10
None at all									A great deal
How much knowledge do you feel you have now on this topic?									
1	2	3	4	5	6	7	8	9	10
None at all									A great deal
How much do you think today’s session has helped your recovery?									
1	2	3	4	5	6	7	8	9	10
None at all									A great deal
Overall how useful did you find the session?									
Unhelpful		Fairly unhelpful		Unsure		Fairly helpful		Very helpful	
How anxious do you feel?									
1	2	3	4	5	6	7	8	9	10
None at all									A great deal
